# Regulation of Inflammatory Reaction in Health and Disease

**DOI:** 10.3390/ijms22105277

**Published:** 2021-05-17

**Authors:** Massimo Fioranelli, Maria Grazia Roccia, Dana Flavin, Linda Cota

**Affiliations:** 1 Department of Human Sciences, Guglielmo Marconi University, 00193 Rome, Italy; mgrroccia@gmail.com; 2 Instituto Terapie Sistemiche Integrate, 00181 Rome, Italy; dana_fk@hotmail.com (D.F.); lindacota77@gmail.com (L.C.)

**Keywords:** low grade chronic inflammation, low dose medicine, psycho-neuro-endocrine-immunology, systems medicine (SM)

## Abstract

Inflammation is a key mechanism for the clearance of infective agents and other inflammatory triggers and is pivotal for the repairing processes of the affected tissues. Inflammation is a multistep process driven by a great number of mediators which regulate specific aspects of the inflammatory response, in agreement with a well-defined chronobiological program. A great number of inflammation-related diseases show a deeply altered immune chronobiology (e.g., COVID-19-related cytokines storm). This aspect highlights the need for a deeper understanding of the inflammatory phenomenon. It is fundamental to study inflammation as a multilevel phenomenon. Of particular interest is the low-grade chronic inflammation, which is an etiological factor of many chronic diseases. Nowadays, the therapeutic approach to low grade chronic inflammation is one of the great challenges of traditional pharmacology. Currently, no drugs specifically designed for the treatment of chronic inflammatory forms are available. Today, bioregulatory systems medicine (BrSM) and low dose medicine (LDM), two pharmacological paradigms grounded in systems medicine, potentially represent new tools for the treatment of inflammation-related diseases. Scientific research has assessed the effectiveness and safety of both these therapeutic approaches, in particular for the management of chronic inflammatory conditions and chronic immunological dysregulations.

## 1. Introduction

For more than two thousand years, it has been known that the inflammatory phenomenon, resulting from an infection or wound, represents a defensive strategy. It is a fundamental mechanism for the clearance of the infectious agent and repairing the affected tissues. The goal of the inflammatory process is the full recovery of the state of good health and the return to conditions of homeostasis.

Aulus Cornelius Celsus (30 BC–38 AD), in “De Medicina”, was the first to clearly describe the cardinal signs of the acute inflammatory process, i.e., rubor, calor, tumor, and dolor, to which Galen (130–200 AD) added the sign functio laesa [1]. The evolution of medical knowledge has led, over the centuries, to define the biological mechanisms that support the inflammatory process in an increasingly in-depth way. In the 1960s, the physiological meaning of the signs described by Celso was clarified: Rubor: vasodilation; Calor: local increase in temperature due to hyperemia; Tumor: exudation; Pain: stimulation of nerves caused by local tension and pH lowering [2].

Following the historical evolution of the study of the inflammatory process, the centrality that has always been attributed to this phenomenon is clear. For a long time, inflammation has been seen exclusively under a totally negative light, a pathological condition that needs to be completely suppressed. This approach, however, clearly contrasts with the physiological function of the inflammatory process. Is symptomatic treatment of inflammation always necessary? Does it occur immediately after signs and symptoms appear?

The limit between the positive and negative valence of a symptom, the border that separates the symptom as a suitable physiological manifestation from becoming a negative pathological sign, is its persistence and/or the supervening lack of self-bioregulation by the organism and its homeostatic control systems.

These two elements make a manifestation of human physiology the sign of a disease [3].

The clinical consequence is that the term inflammation describes a variety of biological processes, with important reflexes on inflammation symptoms treatment. Knowing if an anti-inflammatory drug has positive or negative effects on patient’s healing response is difficult.

In summary:

- inflammation is not a single process [2],
- it is not binary in nature (‘‘on’’ or ‘‘off’’), but it can be regulated by many factors in the cell’s environment [2].

Purpose of this paper is to show how, with innovative pharmacological approaches, is possible to modulate the immune response in an effective way. This approach opens intriguing new opportunities in the management of pathologies with purely inflammatory etiology and with a marked dysregulation component of the immune response.

## 2. Brief Overview on the Role of Main Cytokines in Inflammation

Inflammation is a multistep process currently recognized to be due to an accumulation of leukocytes, mast cells and platelets, which release various types of lipid mediators (eicosanoids), protein (cytokines and chemokines), and gaseous mediators (nitric oxide, carbon monoxide, reactive oxygen species) to circumscribe the affected site, lead off the immune response, eliminate the triggering factor, and thus restore the state of physical integrity.

Each of these mediators regulate specific aspects of the inflammatory response (albeit with a certain degree of redundancy), which is why they are typically recognized as potential pharmacological targets in an inhibitory sense.

Main inflammatory triggers, such as microbial and viral degradation products and cytokines, e.g., interleukin-1β (IL-1β), interleukin-6 (IL-6), and tumor necrosis factor-α (TNF-α), determine the onset of mediate inflammation through interaction with the Toll-Like Receptors (TLRs), IL-1 receptor (IL-1R), IL-6 receptor (IL-6R), and the TNF receptor (TNFR) [4].

Receptor activation modulates intracellular signaling pathways, including the ones controlled by Mitogen-Activated Protein Kinase (MAPK), Nuclear Factor kappa-B (NF-κB), and Janus Kinase (JAK)-Signal Transducer and Activator of Transcription (STAT). These transcription factors promote the expression of cytokines, modulating a great number of inflammatory genes, such as IL-1, TNF-α, IL-6, Interferons, Transforming Growth Factor (TGF), and chemokines. The dysregulation of NF-kB, MAPK, or JAK-STAT derived signal pathways is an important etiologic factor for many inflammatory, autoimmune, and metabolic diseases and it is responsible for the complex alteration of the chronobiology of the immune response during the entire duration of the inflammatory event [4].

## 3. SARS-CoV-2 Pandemic: An Inflammatory Challenge

With the advent of the SARS-CoV-2 pandemic, the alteration of immune response chronobiology was recognized as a key aspect to understand the role of signaling molecules, mainly mediators of inflammation, within the so-called cytokine storm. This is a complex acute immunological event, pivotal in the onset and progression of the Coronavirus Disease 19 (COVID-19) and marker directly linked with disease severity. SARS-CoV-2 infection promotes a series of physio-pathological responses that involves the main inflammatory mediators. The overexpression of proinflammatory cytokines, such as IL-1, IL-6, IL-12, Interferon gamma (IFN-γ), and TNF-α, preferentially targets lung tissue but it is also responsible of progressive multi organ failure, typical of severe cases of COVID-19 [5].

It is also important to highlight that acute inflammation is not the only form of response linked with SARS-CoV-2. Low grade chronic inflammation plays a key role in Long-COVID sequelae, which is a not fully understood disease condition characterized by health problems for more than 12 weeks.

The symptoms of long-COVID can vary widely, but it is quite clear that the neurological symptoms (affecting about 50% of post-COVID patients) are strongly linked. A persistent challenge to the immune system, low-grade neuroinflammation, mainly driven by IL-1, IL-6, and TNF-α, causes cognitive decline, depression, and persistent fatigue [6].

This brief excursus on the main mechanisms that regulate the inflammatory response, and the need for a deeper understanding of the inflammatory phenomenon related to SARS-CoV-2 pandemic highlight the need for a paradigm shift. It is time to shift from the study of the inflammatory response as a phenomenon to a closer study of its systemic reflexes. Therefore, it is fundamental to analyze inflammation, as a multilayer phenomenon, at clinical, cellular, and molecular levels.

## 4. PNEI and Systems Medicine for the Study of Inflammation’s Mechanisms

Inflammation can be considered the paradigm of what has been stated: it is a defense process guided by biological molecules (neuropeptides, hormones, cytokines, growth factors). Therefore, it is physiological and positive. When does it lose its positive connotation to become a pathology itself? When do its signs lead to functio laesa? This shift occurs when physiological resources (autologous anti-inflammatory molecules) are unable to naturally “resolve” the inflammatory symptoms. It is precisely at that moment that the “good” symptom becomes a “bad” symptom. It is at that moment that corrective pharmacological intervention is required.

Failure to use it would induce the progression of the pathology, the onset of functional and structural damage and the onset of a chronic inflammatory process characterized by the absence of signs and symptoms, a low intensity chronic inflammation: low grade chronic inflammation (LGCI) [7]. LGCI is at the same time cause and effect of many chronic diseases that are widespread and often with a fatal outcome [8,9,10,11,12,13,14,15,16,17,18]. The comprehension of the mechanism of LGCI is fundamental for correct diagnosis and treatment.

Until the 1970s, the inflammatory process was studied above all in its pathological value, investigating its biochemical mechanisms and intervening therapeutically by blocking the mechanisms themselves. Subsequently, inflammation was interpreted as a purely immune phenomenon, neglecting its overall vision again.

Starting from the second half of the 1970s, the development of psycho-neuro-endocrine-immunology (PNEI: discipline that studies the interrelationships between the functioning of the nervous system, the immune system, and the endocrine system) by R. Ader [19] offered the possibility and the tools to study the inflammatory phenomenon in its complexity and to identify the PNEI mechanisms that regulate all the phases of the inflammatory phenomenon, from the onset to its resolution.

The control over the organic functions, and therefore over the inflammatory parameters, is mainly exercised by the central nervous system and the neurovegetative system, the endocrine system, and the immune system which, together, constitute the PNEI network.

PNEI vision paved the way to the modern interpretation of the inflammatory phenomenon [20,21]: A perfect physiological mechanism guided by neuro-immuno-endocrinologically and metabolically controlled.

Inflammation is such a physiologically indispensable process that the body itself wants it, triggers it, develops it, turns it off.

Similarly, to PNEI view, and taking origin from the conceptual basis of systems biology, systems medicine (SM), also called network medicine, studies the organism as a network of interconnected networks with self-organization capacity operated through mechanisms of self-regulation, intrinsic stability, robustness, resilience that refer to the concept of homeostasis according to the concepts theorized by the scientist von Bertalanffy [22,23,24,25,26,27]. SM considers disease as a dysregulation of biological networks that dynamically changes throughout the evolution of the disease process and with the development of comorbidities. The robustness of the networks indicates their ability to counteract the dysregulations during the phases of perturbation, returning to the state of stability or ensuring the best possible stability through compensation mechanisms. The treatment of dysregulated networks before the phenotypic symptomatologic outcomes offers the possibility to treat and prevent pathologies in the preclinical phase and potentially reverse the pathological process, stop it, or prevent comorbidities.

The immune-inflammatory network is one of the four fundamental functional networks whose balance determines the maintenance of the conditions of physiological homeostasis [28].

## 5. Low Grade Chronic Inflammation (LGCI) in Diseases

**Figure 1:**
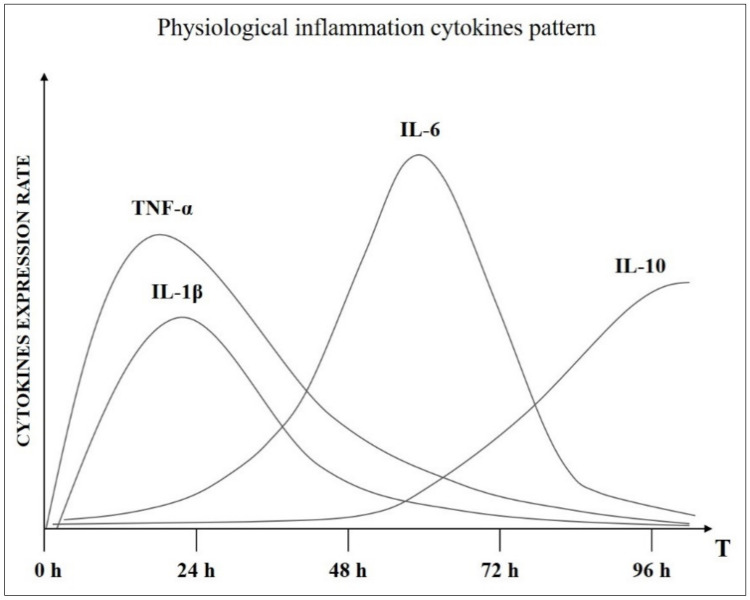
Cytokines chronobiological pattern in normal inflammatory condition.

The physiological status of an organism coincides with the condition of homeostasis/allostasis, in which the vital parameters (fundamentally: pH, body temperature, blood sugar levels and partial pressure of oxygen) are kept within a precise and defined range and whose upward or downward deviation is identified with the pathological state. Inflammation is fully part of the homeostatic controlled physiological functions. Therefore, there is a level of inflammation that falls within the parameters of “normality” defined as physiological inflammation (Figure 1): the mechanism through which the body performs the task of recover or maintaining its own state of health. Maintenance and recovery, two terms with a deep biological meaning:

- **Maintenance:** Even when we are perfectly healthy, inflammatory processes take place in our body. Inflammatory processes must take place because they are the only system to neutralize the stressors that inevitably penetrate the “open system” of the human organism, alternating the PNEI balance. Furthermore, a small inflammatory level is, for example, necessary at the level of the intestinal mucosa to ensure the function of the intestine and ensure the necessary immunotolerance. All this happens automatically and, above all, asymptomatically.
- **Recovery:** When the pro-inflammatory power of a stressor exceeds the control capacity of the defensive systems, the body is forced to amplify its inflammatory response. It is only at this point that the inflammatory symptoms appear, and it is at this moment that the inflammation acquires a pathological value.

**Figure 2:**
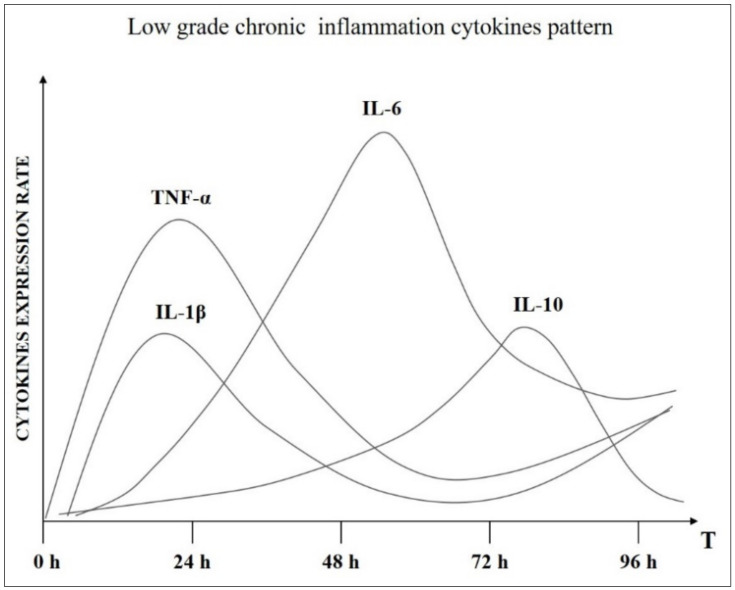
Altered cytokines chronobiological pattern in chronic inflammatory condition.

If the processes described are both physiological, when does the inflammatory phenomenon take on the connotation of LGCI? There are two key fundamental aspects that need to be evaluated: the timing of the phenomenon and the chronobiological intervention of the cytokines that support it [29]. The LGCI presents a series of peculiarities that outline its pathological profile (Figure 2):

1. Timing: The two classic phases of inflammation, maintenance, and recovery, coexist temporally, the inflammation is continuously fed and the restitutio ad integrum phase is not completed.
2. Chronobiology of cytokines: The sequential release phases of cytokines are altered, so IL-1, TNF-α, and IL-6 in order are no longer down-regulated effectively. Levels of these cytokines about 3–4 times higher than baseline are recorded well beyond the 72–96 h typical of acute inflammation. At the same time, there is no up-regulation of anti-inflammatory IL-10. In practice, the inflammatory stimulation lasts over time.

The danger of LGCI is therefore twofold, on the one hand the continuous pro-inflammatory triggering compromises the efficiency of the body’s immune response, exhausting its ability to react, on the other hand the endocrine-metabolic alteration that consequently turns out to be a substrate for the onset of the numerous inflammatory pathologies.

**Figure 3:**
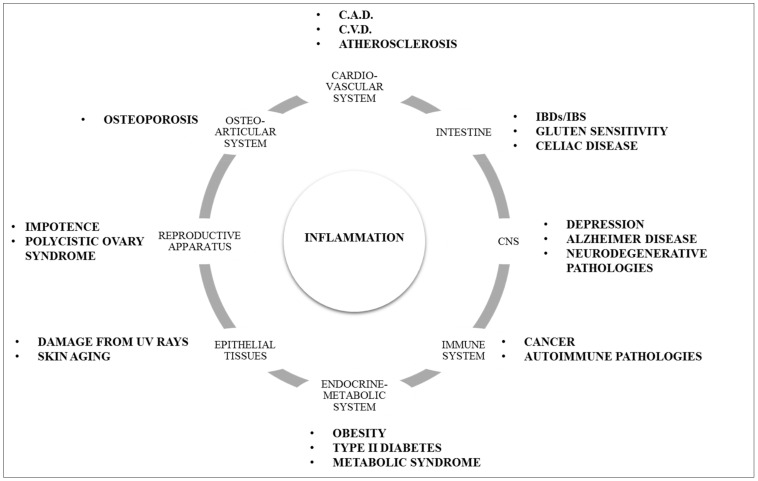
Correlation between inflammation and some chronic diseases.

The LGCI is a characteristic element of many chronic diseases, often with a bad outcome (Figure 3). Subacute levels of circulating IL-1, TNF-α and IL-6 have been observed in obese subjects, in smokers and in patients with type II diabetes. They are also fundamental markers for assessing the risk of myocardial infarction, they are linked to the onset of neurodegenerative diseases and depressive syndromes, their expression is altered in metabolic diseases such as osteoporosis [30]. The main mechanism of LGCI onset is based on a central point, that is the nuclear translocation of the transcription factor NF-kB. The activation of transcription and translation of specific pro-inflammatory genes is manifested by the release of the cytokines in question, IL-1, TNF-α, and IL-6. At the same time, the oxidative damage produced by the inflammatory trigger reduces the production of AMP-kinase (AMPK), a fundamental inhibitory control factor of NF-kB translocation. The negative control function on the inflammatory cascade is thus lost. Without more negative feedback on the starter of the inflammatory cascade, the functional recovery mechanisms cannot be activated, and the inflammation feeds itself, becoming chronic.

## 6. The Traditional Approach to Chronic Inflammation Management

The therapeutic approach to chronic inflammation is one of the great challenges of traditional pharmacology. Currently, drugs designed for use in acute cases and for the treatment of chronic inflammatory forms are used. This is a makeshift solution that unfortunately does not respond to real therapeutic needs. It is known that the chronic use of drugs studied for the acute not only does not allow the achievement of the therapeutic goal but also expose the patient to a series of adverse events due to the use for incongruously long times of drugs studied for acute use for previous periods. Paradigmatic is the use of Piroxicam [31] in the treatment of rheumatoid arthritis, the experimentation of which has shown that not only is it not effective in the treatment of the inflammatory component but is also linked to a worsening of the normal course of the disease from a histological point of view. These results have once again highlighted how the hypothesis regarding the chronic use of NSAIDs must be further studied.

## 7. Low Dose Drugs for the Management of Acute and Chronic Inflammation

As widely explained, inflammation represents, almost paradigmatically, an example of biologically appropriate defensive response, implemented by the body against a pathogenic noxa.

The complexity of the inflammatory phenomenon, especially chronic, requires equally advanced study tools capable of integrating its various levels (clinical, cellular, and molecular). To date, systems medicine (SM) represents the only tool capable of studying a complex problem such as inflammation and its modulation.

SM helps the MDs to overcome the reductionist perspective, which represented the guiding paradigm of medicine in the last century by addressing the clinical approach to the patient and the development of new drugs. According to the reductionist viewpoint, a complex system such as the human organism is divided into multiple parts, considered in isolation or as individual parts of a whole in which, from time to time, a particular relevance and importance is attributed to a single part higher than the others. This was the case in the interpretation of physio-pathology and in the investigation of the etiopathogenetic pathways of most diseases, as well as in the research and development of drugs, which rarely deviate from the one-drug-one cell-one target guideline (a drug-a cell-a target) and which have almost always been designed to act on a single target without considering its multiple connections and interrelationships, coming to idealize them as a sort of magic bullet.

Today, two pharmacological paradigms, grounded in SM, named (i) bioregulatory systems medicine (BrSM—based on the use of low dosages of natural principles mainly of vegetal, mineral, chemical and animal origin combined in multicomponent/multitarget drugs) and (ii) low dose medicine (LDM—based on the use of low dosages of biological molecules such as cytokines, neuropeptides, hormones, and growth factors) have provided the MDs new tools for the treatment of inflammation-based diseases.

In recent years, basic research has clarified the mechanism of action of low doses, and, at the same time, clinical research has supported the validity of this innovative pharmacological approach to inflammatory pathology with the evidence of results deriving from controlled studies. Of fundamental importance in understanding the mechanisms of regulation of inflammatory response was the model of the immune balance, that is, the reciprocal relationships between some lymphocyte subpopulations, T-helper 1 (Th1), Th2, regulatory T cells (T-reg) and Th17. Preclinical and clinical studies on the use of multicomponent/multitarget drugs [32,33,34,35,36,37] and physiological low dose cytokines [38,39,40,41,42,43] have opened new scenarios on the possibilities of treating acute and chronic inflammatory diseases (also on an autoimmune basis).

Acting on the inflammatory process by working on the profound etiological cause underlying the immune-inflammatory process appears possible today. BrSM multicomponent/multitarget drugs and SKA (Sequential Kinetic Activation) activated low-dose cytokines can recover the balance between lymphocyte subpopulations through cross-regulation mechanisms through the up- and down-regulation of the trans-membrane receptors of the different Th lymphocytes.

To work effectively on the inflammatory phenomenon through the modulation of the cytokines involved, it is essential to know the exact chronology with which the inflammation mediators are released and act. Classic anti-inflammatory therapy focuses attention on these secondary mediators: Nonsteroidal anti-inflammatory drugs (NSAIDs) block Cyclooxygenase-2 (COX2), cortisone blocks Prostaglandin E2 (PGE2), and acetylsalicylic acid (ASA) acts on the release of nitric oxide (NO). It appears evident that the direct modulation of IL-1 represents a fundamental point to overcome this approach. It clearly emerges that a fine regulating action on the activity of IL-1 and IL-10, for example, can be exploited for the management of inflammation. In particular, the blockade of IL-1 allows to control the acute inflammatory phenomenon, acting on one of the first motors of the signal cascade. The stimulation of IL-10, on the other hand, represents an excellent opportunity to control acute and chronic inflammation where, as already mentioned, the repair process cannot overcome the inflammatory one. By increasing the anti-inflammatory signaling of IL-10, the two phases are rebalanced. Similarly, the therapeutic use of Transforming Growth Factor-β (TGF-β) provides support to the inflammation resolution processes and promotes restitutio ad integrum. The supporting function of the inflammatory process implemented by IL-6 can be counteracted using an opposing cytokine such as IL-4 both in the acute phase and, for example, for the contrast of the triggers of autoimmune diseases.

**Figure 4:**
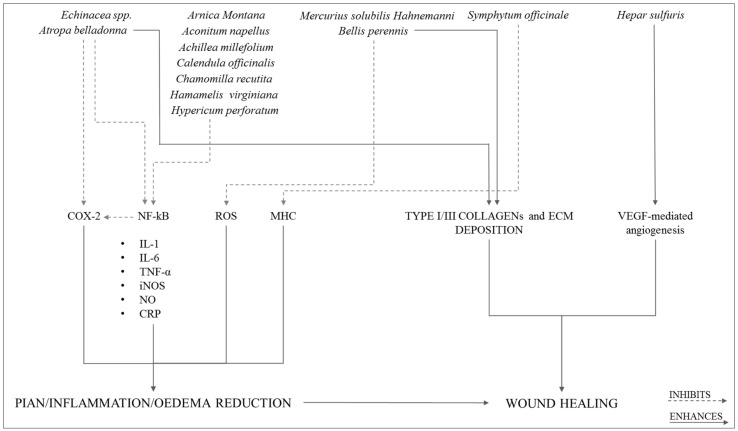
Synoptic diagram of the biological actions performed by each component of Tr14.

With the identification of lipoxins, molecules produced by omega-6 Polyunsaturated fatty acids (PUFAs), resolvins, protectins and maresins, produced by omega-3 PUFAs, today we begin to concretely explore both the biological mechanisms underlying the active resolution of the inflammatory process and possible implications positive that the control of this phenomenon can have for the treatment of inflammatory diseases [44,45,46,47]. A recent research [33] looks very intriguing, specifically the innovative description of the low dose multicomponent natural medication Tr14 (12 herbal extracts and 2 minerals), able to both control the inflammatory signs and induce the pro-resolution phase of the inflammation (Figure 4).

**Figure 5:**
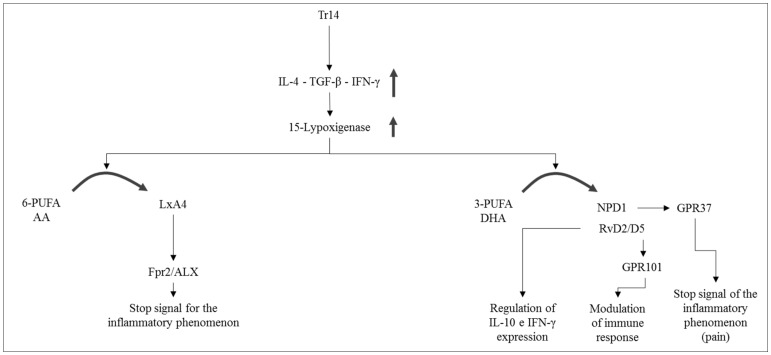
Interleukin-4, TGF-beta and IFN-gamma are physiological regulators of lipoxygenase expression and suggest an important link between 15-lipoxygenase function and the immune/inflammatory response.

By virtue of its composition [48,49,50,51], Tr14 also able to actively promote the expression of the following Specialized pro-resolving mediators (SPMs): (i) lipoxin A4 (LXA4); (ii) Resolvin D2-D5 (RvD2/D5); (iii) Neuroprotectin D1 (NPD1) https://patents.google.com/patent/WO2020089205A1/en, accessed on 1 February 2021 (Figure 5).

Unlike other anti-inflammatory drugs, such as acetylsalicylic acid, Tr14 does not control the expression of SPMs through the COX-2 acetylation pathway (which blocks the physiological inflammatory pathway) but, instead, acts through immunological mediators, such as IL-4, TGF-β, and IFN-γ, signaling molecules active in the control of the enzymatic cascade that lead to the synthesis of SPMs. The expression of one of the key enzymes, 15-lipoxygenase (15-LO) is positively controlled by IL-4, TGF-β, and IFN-γ (Figure 5).

The transcriptomic studies performed on this compound showed its ability to shift M1 to M2, activating the process mediated by lipoxins and resolvine, finally resulting in the inflammation resolution.

Since macrophages are key elements in the immune response, their activation is reflected on the other components of the immune system and is in turn affected by them.

The term “macrophage activation” was introduced in 1960 by Mackaness to indicate the triggering of its antigen-dependent non-specific microbicidal activity. This mechanism was then defined as “classical activation” linked to the action of Th1-derived mediators (e.g., IFN-γ). Subsequently, the so-called alternative pathway of macrophage activation guided by Th2-derived mediators (e.g., IL-4) was identified. The two pathways took the name of activation M1 and M2 for parallelism with the Th1 and Th2 stimuli that drive them.

Over time, other mediators have been added as drivers of the respective activation responses: M1 activators are also TNF-α, Lipopolysaccharides (LPS) and Lipoteic Acid (LTA) viruses, and Granulocyte-macrophage colony-stimulating factor (GM-CSF), while Immunoglobulin G (IgG), IL-10, glucocorticoids, and Macrophage colony-stimulating factor M-CSF push the alternative M2 pathway [52,53].

It is important to note that the two ways are both activation and not one suppressive of the other. Moreover, in inflammatory processes their activation is sequential, first M1 and then M2. The M1-M2 shift is in fact fundamental: a correct passage from the pro-inflammatory M1 phenotype to the M2 anti-inflammatory one, which accelerates the processes of resolution of the inflammatory phenomenon.

## 8. Low Dose Medicine and Scientific Research

In recent years, the scientific research in the field of BrSM and low dose medicine have demonstrated the validity of the conceptual approach and the effectiveness and safety of therapeutic intervention based on the oral administration of multicomponent/multitarget drugs and sub-nanomolar low doses of signaling molecules for the modulation of the inflammatory process [29,30,31,32,33,34,35,36,37,38,39,40].

Some of the studies, especially the clinical ones, deserve to be briefly analyzed to highlight how multicomponent/multitarget and low dose SKA medicines are effective and safe both when compared with traditional medicines, and combined with them in complex therapies in the fields of inflammation and immune reaction management.

For example, Dr. Gonzalez de Vega and colleagues performed a prospective, multicenter, randomized, blinded, active-control and non-inferiority study [36] involved 449 physically active adults sustaining unilateral grade 1 or 2 ankle sprain within the past 24 h. More specifically, 152 subjects were randomized to receive 2 g of Tr14 ointment; 147 subjects received diclofenac gel both medications were administered topically to the ankle three times a day for 14 days, with six weeks follow up. Obtained results highlighted the non-inferiority of Traumeel vs. diclofenac for reducing pain and functional improvement. At six weeks, all participants reported total pain relief and normal functioning. Treatments were equally well tolerated. In conclusion, both Tr14 and Diclofenac decreased pain and improved joint function in acute ankle sprain and were well tolerated.

Another work conducted on Tr14 and Ze14 demonstrated the efficacy of these multitarget/multicomponent medicines in inflammatory pain management. Dr Lozada and colleagues evaluated a combination of dilute biological and mineral extracts (Tr14 and Ze14) administered IA for painful knee Osteoarthritis (OA). In this multi-center double-blind, randomized, controlled trial (db-RCT) [37] 232 patients with moderate-to-severe chronic knee OA were randomized to 3 weekly IA injections of either Tr14 & Ze14 or saline. The primary efficacy outcome was change in knee pain from Baseline to end-of-study, measured by the WOMAC OA Pain Subscale. Secondary outcomes included Total WOMAC and subscores for stiffness and physical function, change in pain following 50 feet walk and physician global assessments. Clinical relevance was assessed by comparing proportions of patients with reductions from baseline in WOMAC scores greater than a validated benchmark Minimal Clinically Important Difference (MCID). Safety was assessed by monitoring of vital signs, physical examinations of the target knee, adverse events, and concomitant medications. Tr14 & Ze14 provided statistically significant and clinically relevant pain relief in comparison to placebo. In this double-blind, randomized, controlled trial, a biological/mineral multi-extract combination was shown to be a safe and effective treatment for pain in moderate-to-severe knee OA.

In the field of inflammatory pain management and control of disease activity, Dr. Martin-Martin and colleagues performed a prospective, open, randomized, active-controlled study [40] on the effects of oral administration of low dose SKA cytokines of IL-4, IL-10, and low dose SKA anti-IL-1 antibodies in patients with Rheumatoid Arthritis. Patients, after treatment with anti-TNF-α monoclonal antibodies, were randomized into two experimental groups and received low dose therapy (Group A) for 12 months or biological therapy with disease-modifying antirheumatic drugs (DMARDs) (Group B) for 12 months, to maintain a low disease activity rate. The results showed the non-inferiority of the low dose treatment compared to the conventional one with DMARDs. The preliminary data obtained, although preliminary, show how the therapy based on Low Dose SKA molecules can be advantageously used for the therapy of rheumatoid arthritis and to maintain the remission of the disease. The results obtained from this clinical study show that: (i) the use of low-dose SKA cytokines/antibodies administered simultaneously via the oral route showed good efficacy in maintaining low diseases activity (LDA) in subjects with Rheumatoid Arthritis, following remission obtained with biologics or DMARDs; (ii) the difference between the two groups can be observed from the sixth month of treatment from the start of remission, when the group treated with low dose SKA cytokines/antibodies maintains the clinical signs consistent with remission in a homogeneous manner, whereas the group treated with DMARDs gradually deteriorates, with a vast range of intra-group variability; (iii) The study suggests that therapy with Low Dose SKA cytokines/antibodies may act by inducing a progressive regulation and stabilization of the Immune System, justified by the more limited dispersion in the Group treated with low-dose SKA cytokines/antibodies; (iv) the safety was confirmed as being excellent and no adverse event was reported amongst treated subjects.

Also, allergic diseases show an inflammatory component during their onset and progression, due to the Th1/Th2 immune imbalance. Dr. Carello and colleagues studied the efficacy of a treatment with low dose SKA cytokines in a pediatric population suffering from chronic atopic dermatitis. The two-stage double-blind randomized controlled trial [41] included children with mild-to-moderate atopic dermatitis, evaluated using Scoring Atopic Dermatitis (SCORAD) index (score range from 6 up to 40) At the time of enrollment, all children had to present an acute phase of the disease. Children with both IgE and non-IgE mediated atopic dermatitis were included. The primary outcome was the reduction in the severity of atopic dermatitis through the SCORAD index with an expected improvement percentage of 30%. As secondary outcomes, the following were taken into consideration: length of the disease-free interval, tolerability and compliance of treatment and management of adverse events. The results show that the group treated with Low Dose SKA cytokines shows a mean decrease in the SCORAD of 54%, a decrease that continues in the follow-up reaching 64%. In the same observational period, the treated group showed a significant reduction in the medications taken for symptomatic control (antihistamines and topical corticosteroids). The study also highlighted a progressive improvement in quality of life (itching and nocturnal disturbances) of subjects treated with low dose SKA cytokines throughout the investigation period. To conclude, this study suggests that low-dose medicine represents an efficacious therapeutic option for the clinical treatment of atopic dermatitis.

All the works conducted so far show the ability of multicomponent/multitarget drugs and low dose SKA signaling molecules to modulate immune response in a highly selective manner. In particular, the immunostimulating and immunomodulating properties of the tested compounds are clearly described. The ability to act in a refined way on the Th1/Th2 balance is crucial for the management of diseases characterized by an inflammatory component.

## 9. Conclusions

Years of scientific research on BrSM and low dose medicine have allowed scientists to provide scientifically relevant data that can demonstrate:

1. The validity of the theoretical concepts underlying the BrSM/LDM approach, strongly linked with the chronobiology of the inflammatory event and the main immunomodulation pathways.
2. The effectiveness multicomponent/multitarget drugs and SKA signaling molecules and their immunomodulatory and Immunostimulating capacity.
3. The safety of the tested preparations.

From an overall analysis of the data described and those contained in the studies cited, the ability of low dose medicine and BrSM medicines to modulate immune response systems, while respecting their physiological functions, is clear. These evidences trace a new path for the management of problems, especially inflammation-related one, often very complex, for which the classic pharmacological intervention can show limits of safety and compliance.

This is especially evident concerning the pro-resolution phase of the inflammatory phenomenon and the simultaneous restitutio ad integrum, often seen as a spontaneous event temporally secondary to the inhibition of the inflammatory response and not consequent to the physiological modulation of the inflammatory response itself.

Moreover, the particular propensity of low dose and BrSM drugs to control inflammatory conditions and chronic immunological dysregulations allows us to include some of these drugs among the potential tools for the management of LGCI, such as the long-COVID syndrome, which is currently difficult to manage due to its particular symptomatologic complexity.
